# *ESR1* mutations in ER-positive breast cancer: from endocrine resistance to ctDNA-guided therapeutic interception

**DOI:** 10.37349/etat.2026.1002375

**Published:** 2026-05-26

**Authors:** Thais Martinez, Samantha Wegner, Hisham F. Bahmad

**Affiliations:** IRCCS Istituto Romagnolo per lo Studio dei Tumori (IRST) “Dino Amadori”, Italy; ^1^Herbert Wertheim College of Medicine, Florida International University, Miami, FL 33199, USA; ^2^Campbell University School of Osteopathic Medicine, Lillington, NC 27546, USA; ^3^Department of Pathology and Laboratory Medicine, University of Miami Miller School of Medicine, Miami, FL 33136, USA

**Keywords:** *ESR1*, estrogen receptor, breast cancer, endocrine resistance, ctDNA, review

## Abstract

Endocrine resistance in estrogen receptor-positive (ER+) breast cancer has undergone a fundamental reconceptualization over the past decade. The discovery that activating mutations in the *ESR1* gene encoding ERα emerge under aromatase inhibitor (AI) selection pressure and drive ligand-independent receptor activation established a shift from empirical treatment sequencing to molecularly guided intervention. This review provides a synopsis of the structural biology underlying constitutive ER activation, the evolutionary dynamics of *ESR1*-mutant clones detectable through circulating tumor DNA (ctDNA), and the clinical evidence demonstrating that early molecular detection can trigger therapeutic switches that alter disease trajectory. The regulatory approval of elacestrant for *ESR1*-mutant disease and randomized trial data showing progression-free survival (PFS) benefit from ctDNA-guided endocrine switching (PADA-1, SERENA-6) position *ESR1* genotyping as a dynamic biomarker with direct therapeutic implications. We examine the integration of oral selective ER degraders (SERDs) into treatment algorithms, the role of co-occurring alterations in the phosphatidylinositol 3-kinase/protein kinase B (PI3K/AKT) pathway, and emerging directions, including machine learning approaches to ctDNA kinetics and adaptive trial designs that treat clonal evolution as an actionable target. The convergence of structural mechanisms, liquid biopsy technology, and biomarker-driven drug development provides a framework for precision oncology in endocrine-resistant breast cancer. While these advances are substantial, important challenges remain, including the lack of mature overall survival (OS) data from interception trials, cost and accessibility barriers to serial ctDNA monitoring in diverse global healthcare settings, the unresolved question of optimal therapeutic sequencing in patients with concurrent *ESR1* and PI3K pathway alterations, and the need to distinguish clinically actionable low-variant allele frequency (VAF) *ESR1* calls from background noise in liquid biopsies.

## Introduction

Endocrine therapy remains the cornerstone of treatment for estrogen receptor-positive (ER+) breast cancer. By suppressing or antagonizing ER signaling, hormonal endocrine therapy offers an effective and less toxic systemic approach for breast cancer patients. However, resistance to hormonal therapy is common, and metastatic ER+ breast cancer continues to cause high cancer-related mortality rates globally [1, 2]. For decades, endocrine resistance was believed to be mainly due to loss of ER expression, pathway bypass mechanisms, or nonspecific cellular adaptation. This paradigm shifted fundamentally with the application of next-generation sequencing (NGS) to metastatic tumor specimens.

Multiple independent genomic analyses identified recurrent, activating mutations in the *ESR1* gene encoding ERα, in patients with endocrine-resistant metastatic disease [1, 3]. These alterations were rare in treatment-naïve primary tumors but became enriched following aromatase inhibitor (AI) exposure, establishing a clear role for therapeutic selection pressure [1, 2]. *ESR1* ligand-binding domain (LBD) mutations stabilize the receptor in a constitutively active conformation, enabling ligand-independent transcriptional signaling even in the absence of estrogen [1, 3]. This mechanism explains why continued estrogen deprivation becomes ineffective once these mutant clones emerge. Importantly, these tumors often retain partial sensitivity to agents that directly antagonize or degrade the receptor, providing a rational therapeutic target [4].

Another paradigm shift followed with the recognition that *ESR1* mutations are readily detectable in circulating tumor DNA (ctDNA). Metastatic ER+ breast cancer frequently involves bone and visceral sites, where tissue sampling can be either challenging or technically compromised by decalcification. ctDNA offered an alternative, blood-based detection that captures tumor heterogeneity across multiple metastatic sites and permits serial monitoring over time [5–7]. ctDNA analysis provided more than a surrogate for tissue testing. Serial monitoring revealed that *ESR1* mutations often emerge months before radiographic progression, with mutant allele fractions rising under continued AI therapy and declining when selective pressure changes [8–12]. These observations reframed resistance as a measurable, dynamic evolutionary process rather than a binary event.

The field then reached a critical milestone: therapeutic intervention based on ctDNA detection. The phase III PADA-1 (palbociclib and circulating tumour DNA for *ESR1* mutation detection) trial demonstrated that detecting a rising *ESR1* mutation in plasma could trigger an early endocrine switch that improved progression-free survival (PFS), even while maintaining cyclin-dependent kinase 4 and 6 (CDK4/6) inhibition [13–15]. Subsequent studies using next-generation oral selective ER degraders (SERDs) have strengthened the concept of molecularly guided interception rather than delayed salvage therapy [16, 17]. Concurrently, drug development has accelerated. Elacestrant became the first oral SERD approved specifically for *ESR1*-mutant metastatic breast cancer, supported by randomized data and regulatory analyses explicitly linking benefit to mutation status [17–23]. Additional agents (including camizestrant, imlunestrant, and proteolysis-targeting chimera (PROTAC)-based ER degraders such as vepdegestrant) have expanded the therapeutic armamentarium and raised new questions regarding optimal sequencing, combination strategies, and timing of intervention [16, 24–27].

**Figure 1:**
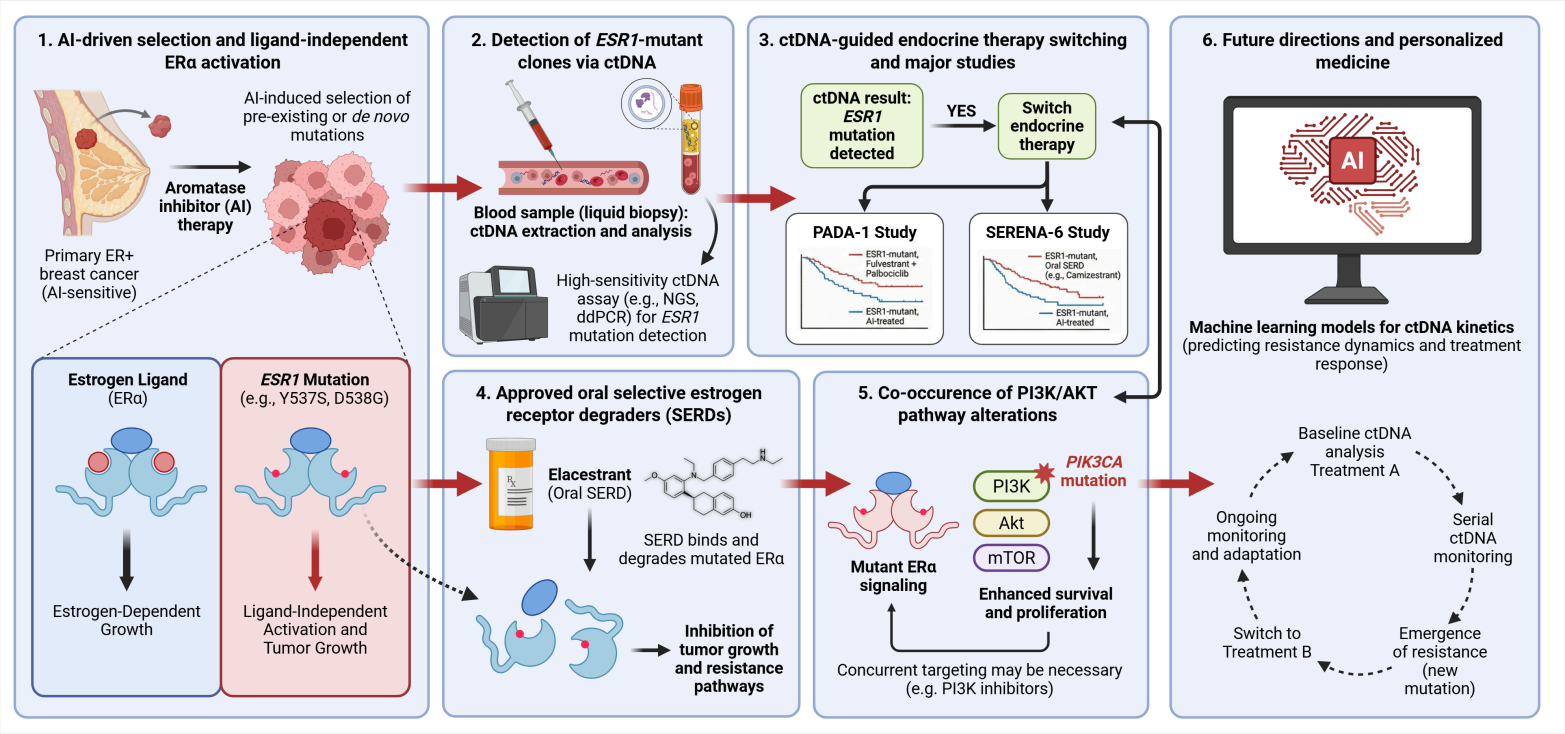
**From AI-induced selection to ctDNA-guided therapeutic interception in *ESR1*-mutant ER+ breast cancer.** (**1**) Prolonged AI therapy exerts selective pressure that enriches pre-existing or de novo *ESR1* ligand-binding domain mutations (most frequently Y537S and D538G), enabling ligand-independent ERα activation and continued tumor growth despite estrogen deprivation. (**2**) Emergent *ESR1*-mutant clones are detectable noninvasively through ctDNA via high-sensitivity assays (ddPCR or targeted NGS), allowing dynamic quantification of VAF. (**3**) Rising ctDNA-detected *ESR1* mutations can trigger endocrine therapy switching prior to radiographic progression; randomized evidence from PADA-1 and SERENA-6 demonstrates that this molecularly guided interception improves PFS. (**4**) Approved SERDs, exemplified by Elacestrant, directly bind and degrade mutant ERα, circumventing ligand-independent signaling and restoring endocrine sensitivity. (**5**) Co-occurring PI3K/AKT pathway alterations (including *PIK3CA* mutations) provide parallel survival signals that may necessitate combinatorial targeting strategies. (**6**) Future directions encompass machine learning-assisted modelling of ctDNA kinetics for resistance prediction and adaptive clinical trial designs that treat clonal evolution as an actionable, prospectively interceptable target. AI: aromatase inhibitor; ctDNA: circulating tumor DNA; ddPCR: droplet digital PCR; ER+: estrogen receptor-positive; mTOR: mechanistic target of rapamycin; NGS: next-generation sequencing; PFS: progression-free survival; PI3K/AKT: phosphatidylinositol 3-kinase/protein kinase B; VAF: variant allele frequency. Created in BioRender. Bahmad, H. (2026) https://BioRender.com/p9oyl08

Together, these advances positioned *ESR1* mutations at the convergence of molecular genetics, liquid biopsy technology, and clinical decision-making [28]. They provided a model for how resistance mechanisms can be detected early, monitored longitudinally, and targeted in ways that alter clinical outcomes. This review examines the biology underlying the clinical relevance of *ESR1* mutations, the evidence supporting ctDNA-based detection and intervention, and the practical considerations required to integrate *ESR1* genotyping into routine clinical practice. Emerging directions are also explored, including artificial intelligence-assisted interpretation of ctDNA kinetics and adaptive trial designs that treat clonal evolution as a therapeutic target (Figure 1). These advances must be considered within the broader context of morphological, radiological, and molecular subtyping challenges in breast oncology.

## *ESR1* mutation biology and the molecular basis of endocrine resistance

In ER+ breast cancer, prolonged exposure to endocrine therapy selects for mutations in the *ESR1* gene, which encodes ERα. These mutations account for a substantial proportion of endocrine resistance, ranging from approximately 36% in earlier reports [3] to 63.2% in more recent cohorts [1, 3, 17, 29]. These mutations predominantly occur in the LBD of ERα and result in ligand-independent receptor activation. This allows tumor proliferation despite estrogen deprivation therapies.

While *ESR1* mutations are rare in untreated primary breast cancer, multiple studies have shown that these mutations become frequent in metastatic disease following exposure to AIs and other endocrine therapies. This pattern suggests that these mutations function as resistance mechanisms rather than primary oncogenic drivers. Among these mutations, Y537S and D538G are the most common, accounting for the majority of *ESR1*-mutant breast cancers [1, 3, 30, 31]. Individual studies report Y537S in 42.3% of cases and D538G in 38.5% of cases [30]. Similarly, Westenend et al. [32] identified D538G in 9 of 18 cases (50%) and Y537S in 7 of 18 cases (39%) in a metastatic cohort. The frequency of these mutations after aromatase therapy further reinforces their role in endocrine resistance. Other substitutions at codon 537, including Y537N and Y537C, were also detected at lower frequencies [30]. For instance, in one metastatic cohort, Y537N accounted for approximately 9% of *ESR1*-mutant cases and Y537C for approximately 4.5% [17]. Although these mutations are less common than Y537S, they still retain their activating properties and contribute to disease progression.

Moreover, mutations affecting codon 536 are reported less frequently but follow the same resistance mechanism. Westenend et al. [32] identified the L536H mutation in 2 of 18 *ESR1*-mutant metastatic tumors, while Venetis et al. [17] detected only a single codon 536 variant in their cohort, supporting the low prevalence of these alterations. Lastly, E380Q represents a rare *ESR1* mutation with a distinct functional profile. This mutation occurs outside the helix 11-12 hotspot region and represents approximately 1.1–10% of *ESR1* mutations depending on the cohort [31, 33–35]. E380Q enhances estrogen sensitivity rather than causes complete ligand-independent receptor activation. Nonetheless, this mutation is detected at lower frequencies across different cancer cohorts, underscoring that not all variants confer resistance through identical mechanisms [1, 31].

Polyclonal mutations are common and clinically significant (Table 1). Multiple *ESR1* mutations are detected in 26–36% of *ESR1*-mutant cases, reflecting parallel evolutionary paths across metastatic sites [17, 36, 37]. This polyclonality has important therapeutic implications, as different *ESR1* variants may respond differently to specific SERDs and other ER-targeting agents [38]. The presence of multiple mutations may necessitate combination therapy approaches rather than single-agent strategies. However, it should be noted that the apparent association between polyclonality and worse outcomes may be confounded by overall ctDNA burden (total mutant allele fraction), as polyclonal *ESR1* mutations are more frequently observed in patients with high ctDNA load, which is itself a strong prognostic factor. After correcting for total *ESR1*-mutant allele frequency (MAF), polyclonality may play a limited independent prognostic role. Indeed, it is plausible that virtually all *ESR1*-mutant cases are polyclonal at a biological level, with minor subclones falling below the detection threshold of current assays.

**Table 1:** Common *ESR1* mutations in metastatic ER+ breast cancer and their functional implications.

***ESR1* mutation**	**Frequency**	**Location**	**Functional consequence**	**Clinical impact**	**References**
E380Q	Less common (1.1–10% of *ESR1* mutations)	LBD (outside helix 12)	Ligand-independent activity; estradiol hypersensitivity; increased DNA binding	Endocrine resistance may be associated with a higher tumor mutational burden, distinct from H11-12 mutations	[31, 33–35]
D538G	Most common in tissue (38–45%); variable in ctDNA	Helix 12, LBD	Constitutive ER activation; retains enhanced estrogen responsiveness; altered chromatin binding	Reduced AI efficacy; retained SERD sensitivity; associated with shorter PFS on exemestane monotherapy (2.69 vs. 3.94 months); may drive CDK4/6i resistance	[17, 30, 39–42]
Y537S	Second most common in tissue (25–42%); variable in ctDNA	Helix 12, LBD	Constitutive ER activation; complete ligand independence; higher coactivator affinity than D538G	Reduced AI efficacy; retained SERD sensitivity; associated with worse OS (19.98 months) than D538G (25.99 months); may drive CDK4/6i resistance	[17, 30, 39–43]
Y537N and Y537C	Common (Y537N ~10%; Y537C ~3–10%)	Helix 12, LBD	Constitutive ER activation; ligand-independent growth	Similar to Y537S; differential drug sensitivities across anti-estrogen classes	[33, 38]
Multiple/Polyclonal	26–36% of *ESR1*-mutant cases	Various	Compound resistance mechanisms; clonal heterogeneity	Complex treatment selection may require combination approaches; variable prognosis depending on specific mutations present	[17, 34, 38]
Both Y537S + D538G	~D538G (21.1%) and Y537S (13.3%) of *ESR1*-mutant cases	Helix 12, LBD	Dual constitutive activation pathways	Reported in a single cohort analysis to have poor outcomes (median OS 15.15 months); requires independent validation; may reflect higher overall ctDNA burden rather than specific mutational combination	[42]

AI: aromatase inhibitor; CDK4/6i: cyclin-dependent kinase 4 and 6 inhibitors; ctDNA: circulating tumor DNA; ER+: estrogen receptor-positive; LBD: ligand-binding domain; OS: overall survival; PFS: progression-free survival; SERD: selective estrogen receptor degrader.

## Clinical consequences of *ESR1* mutations

Clinically, *ESR1* mutations confer resistance to standard hormone therapies such as AIs by promoting ligand-independent activation of the ERs. As a result, tumors with these mutations can grow and progress despite estrogen-lowering strategies [1, 3]. In a genome engineering study, the Y537S mutation demonstrated the highest level of ligand-independent ER activity and required the highest drug concentrations to suppress ER signaling. This suggests that this mutation is frequently associated with greater resistance to anti-estrogen therapies, including Fulvestrant [38]. Mechanically, Y537S stabilizes helix 12 of the ER in its active conformation, making it harder for hormone therapies to turn the receptor off [3]. On the other hand, D538G retains somewhat greater responsiveness to ER degradation compared to Y537S, and treatments such as Fulvestrant remain more effective [38]. Beyond endocrine resistance, *ESR1* mutations have been shown to modulate metastatic behavior, indicating a role in disease progression beyond therapy resistance alone [44]. It is important to note that *ESR1* mutant tumors retain ER expression. As a result, these tumors remain hormonally driven, and endocrine therapy remains a viable treatment strategy. In contrast, tumors with loss of ER expression are associated with a significantly worse prognosis as endocrine therapies are no longer effective [30, 32].

## Detecting *ESR1* mutations: tissue genomics vs. ctDNA

### Why is *ESR1* ideally suited for liquid biopsy?

*ESR1* mutations are acquired alterations shaped by endocrine selection pressure, making them fundamentally different from static genomic biomarkers that can be tested once in primary tissue [7, 8, 14]. These mutations are rare in treatment-naïve primary tumors (< 1–5%) but emerge in 20–40% of patients with metastatic ER+ breast cancer following AI exposure, often appearing as polyclonal events that expand and contract under therapeutic pressure [1, 8, 14]. Metastatic ER+ disease is frequently bone-predominant, making tissue acquisition challenging, uncomfortable for patients, and often yielding compromised DNA quality after decalcification [5, 7, 45]. ctDNA offers critical practical advantages: minimal invasiveness, ability to sample heterogeneity across multiple metastatic sites simultaneously, and capacity for serial monitoring that can detect resistant subclones months before radiographic progression [7, 8, 24]. The plasmaMATCH trial demonstrated that ctDNA testing achieved results in 99% of patients, compared to 70–90% success rates for tissue-based sequencing, with comparable accuracy and faster turnaround times [7].

### Concordance between tissue and ctDNA testing

Concordance between tissue and ctDNA for *ESR1* mutations is high but imperfect, with important clinical implications. In the plasmaMATCH study, ctDNA and tissue showed substantial agreement, though *ESR1* mutations had lower percent-negative agreement, likely reflecting subclonality and ctDNA’s ability to detect mutations present in metastatic sites other than the one biopsied [7]. A meta-analysis of paired tissue-ctDNA studies reported overall concordance of 91% for *ESR1* mutations [46].

Discordance patterns favor ctDNA for *ESR1* detection. In a multicenter analysis of 187 paired samples, ctDNA detected *ESR1* mutations in 18 patients where tissue was negative, while tissue detected mutations in only 3 patients where ctDNA was negative [47]. Among *ESR1*-mutant cases, ctDNA-only detection accounted for the majority of discordant results, with 16 of 29 total *ESR1* mutations detected exclusively in plasma [47]. This pattern reflects both spatial tumor heterogeneity and the acquired nature of ESR1 mutations under endocrine pressure [5, 17].

Importantly, ctDNA negativity does not exclude *ESR1* mutation presence, particularly in patients with low tumor burden, limited ctDNA shedding, or certain metastatic patterns [48, 49]. In such cases, tissue testing from an accessible progressing site remains valuable [17, 45].

### Technical approaches: digital PCR vs. NGS

Two complementary technical strategies dominate *ESR1* testing, each with distinct performance characteristics suited to different clinical questions [5, 17]. Droplet digital PCR (ddPCR) platforms excel at detecting common LBD hotspot mutations (D538G, Y537S/N/C, E380Q) with exceptional analytical sensitivity, achieving limits of detection ranging from 0.07–0.19% VAF [33, 50, 51]. Multiplex ddPCR assays can simultaneously detect 7–17 *ESR1* mutations in a single reaction, providing rapid turnaround (often < 14 days) and robust quantification for serial monitoring [33, 50–52]. In the PADA-1 trial, centralized ddPCR testing of over 12,500 blood samples achieved a median turnaround time of 13 days from blood draw to result notification, with technical failure in < 1% of samples [50]. The primary limitation is coverage, where ddPCR assays target only predefined hotspots and miss rare or novel *ESR1* variants [5, 11].

Targeted NGS panels provide comprehensive coverage of the *ESR1* gene, detecting uncommon variants (E380Q, S463P, V534E, L536R) and simultaneously identifying co-alterations in *PIK3CA*, *AKT1*, *PTEN*, and *ERBB2* that influence treatment selection [5, 53–55]. Modern hybrid-capture NGS assays achieve median unique coverage depths exceeding 7,500×, enabling detection of mutations at VAFs below 1% [53, 55]. However, sensitivity varies with tumor fraction, sequencing depth, and platform design—which is particularly problematic when ctDNA shedding is low [5, 54].

A direct comparison of ddPCR vs. targeted NGS across 200 ctDNA samples showed 84% concordance, with NGS missing 32 cases (16%) with low MAF or insufficient coverage [50]. Conversely, NGS identified rare *ESR1* variants (D538N, P539R) that would be missed by standard ddPCR hotspot panels [54]. In practice, many centers use NGS for initial comprehensive profiling and reserve ddPCR for focused serial tracking when a known *ESR1* mutation is established [5, 11, 55]. In summary, ddPCR offers superior sensitivity and rapid turnaround for focused *ESR1* hotspot monitoring, while NGS provides comprehensive coverage of rare variants and co-alterations; many centers use both platforms in complementary roles.

### Pre-analytical variables and quality control

Pre-analytical factors critically influence ctDNA integrity and assay sensitivity, requiring standardized protocols [56–58]. Blood collection tube selection is the most studied variable: standard K_2_EDTA tubes require processing within 6 hours to prevent white blood cell lysis and genomic DNA contamination, whereas cell-stabilization tubes permit storage up to 48 hours or longer without compromising ctDNA detection [56, 59].

Double centrifugation is essential for isolating cell-free plasma and minimizing genomic DNA contamination [58, 59]. Processed plasma can be stored frozen without affecting downstream ctDNA analysis, but multiple freeze-thaw cycles degrade nucleic acids and should be avoided by aliquoting plasma into single-use fractions [56]. Extraction method influences both cell-free DNA (cfDNA) concentration and size profile, with column-based methods yielding higher total cfDNA but capturing larger fragments, while magnetic bead-based methods recover shorter fragments more efficiently [60].

Reporting standards should enable clinical decisions. For *ESR1*, optimal reports include analytical method, genomic regions covered, limit of detection, VAF (when available), tumor fraction estimate (if applicable), and whether the finding is new vs. persistent [45, 57]. Samples with tumor fraction < 1% should be interpreted with caution, as test sensitivity varies at low tumor content [61, 62]. While samples with tumor fraction < 1% may have reduced sensitivity for comprehensive genomic profiling, the clinical relevance of low-VAF *ESR1* mutations in ER+ metastatic breast cancer under AI therapy has been prospectively validated by both PADA-1 and SERENA-6, where treatment switching based on low-VAF *ESR1* detection yielded significant PFS improvements [14, 15, 24]. Caution regarding low-VAF calls applies primarily to incidental, non-*ESR1* variants of uncertain significance rather than to *ESR1* LBD mutations detected in the appropriate clinical context. In summary, rigorous pre-analytical standardization and comprehensive reporting are essential prerequisites for reliable ctDNA-based *ESR1* testing in clinical practice.

### Clinical timing: progression-based testing vs. molecular interception

Two distinct clinical concepts guide *ESR1* testing timing, reflecting different strategic goals [6, 14, 24]. While the “standard” workflow is older and considered the traditional one, the interception workflow is a newer one:

- **Testing at progression (standard workflow):** American Society of Clinical Oncology (ASCO) guidelines recommend routine *ESR1* testing at recurrence or progression on endocrine therapy (with or without CDK4/6 inhibitors [CDK4/6i]) in patients with ER+/human epidermal growth factor receptor 2 (HER2)– metastatic breast cancer [6, 62]. Testing should be performed on blood or tissue obtained at the time of progression, with blood-based ctDNA preferred owing to greater sensitivity [6]. This approach has become mainstream practice and is supported by high-quality evidence linking *ESR1* mutation status to treatment selection [6, 62].
- **Testing before progression (interception workflow):** This strategy treats *ESR1* emergence as a detectable event that can trigger therapeutic intervention even in clinically stable patients [14, 24]. In PADA-1, serial ctDNA monitoring every 2 months identified rising *ESR1* mutations in 26% of patients receiving AI + palbociclib, occurring most frequently after 6 months and before 3 years of treatment [14, 15, 50]. Patients randomized to switch to fulvestrant upon *ESR1* detection (before radiographic progression) showed improved short-term PFS (hazard ratio [HR] 0.54, 95% confidence interval [CI] 0.38–0.75) and medium-term PFS (HR 0.35, 95% CI 0.22–0.54) compared to continuing the same therapy [14]. Similarly, SERENA-6 demonstrated that camizestrant switching during first-line therapy upon *ESR1* emergence improved outcomes compared to continuing AI [24].

Importantly, the kinetics of *ESR1* mutation emergence during first-line AI + CDK4/6i therapy follow a characteristic bell-shaped temporal distribution. Data from PADA-1 demonstrate that *ESR1* mutations peak between 6 and 30 months of treatment [13, 50]. Mutations are more frequently detected in patients with strongly ER-positive tumors, consistent with greater dependence on ER signaling and thus greater selective pressure from AI therapy. These kinetic observations have important practical implications: serial ctDNA monitoring for *ESR1* should begin no earlier than 6 months into first-line AI + CDK4/6i treatment to optimize detection yield, and monitoring should continue for up to 3 years as late emergence events are well documented [14].

Molecular interception requires operational infrastructure: pre-planned sampling intervals (typically every 2–3 months), clear thresholds for defining “rising” *ESR1* (accounting for assay variability and tumor fraction), and predefined protocols specifying VAF thresholds for calling a rising *ESR1* mutation, the minimum number of confirmatory serial samples, and the timeframe within which a treatment switch should be executed upon molecular detection [14, 37, 63]. Serial monitoring studies show that *ESR1* clearance in subsequent samples correlates with better outcomes, while persistent or early detection (< 6 months) associates with worse prognosis [37, 64].

It is important to note that the standard testing-at-progression workflow represents the established and traditional paradigm, endorsed by the 2023 ASCO Guideline Rapid Recommendation Update as routine clinical practice [6]. The molecular interception workflow, while supported by randomized evidence from PADA-1 and SERENA-6, is a substantially newer strategy not yet formally incorporated into clinical practice guidelines. Although both approaches have clinical evidence supporting their use, they do not currently stand on equal regulatory or guideline footing [13, 15, 50].

The implementation of serial ctDNA monitoring for *ESR1* interception faces significant practical barriers in diverse global healthcare settings. The cost of repeated liquid biopsy testing (whether ddPCR or NGS-based), the requirement for centralized laboratory infrastructure with rapid turnaround times, and variability in reimbursement policies across different healthcare systems all limit widespread adoption outside of clinical trials. In PADA-1, centralized testing was feasible with a median turnaround of 13 days, but this infrastructure is not universally available [50]. The economic differential between focused ddPCR-based *ESR1* monitoring and broader NGS panels represents an important consideration for health systems evaluating implementation strategies. Health economic evaluations comparing the cost of serial monitoring against the potential savings from earlier, more effective therapeutic intervention are urgently needed to support guideline incorporation.

### Interpreting results: common pitfalls and clinical context

Low VAF positives are biologically real but require contextual interpretation. A single low-VAF *ESR1* call may represent early clonal outgrowth, assay noise, or pre-analytical contamination. This depends on tumor fraction, metastatic burden, contemporaneous treatment, and serial trend [5, 50]. A trend across serial samples is more persuasive than a single low-level detection [37, 63].

Of note, clinicians should distinguish between incidental low-VAF variants of uncertain significance (which may warrant cautious interpretation) and *ESR1* LBD mutations detected at low VAF in patients with ER+ metastatic breast cancer receiving AI therapy.

An important consideration in the interpretation of liquid biopsy results is the potential for clonal hematopoiesis of indeterminate potential (CHIP) to confound ctDNA findings. CHIP-associated mutations predominantly affect genes such as *DNMT3A*, *TET2*, and *ASXL1*, and are not typically found in the *ESR1* LBD hotspots (D538G, Y537S/N/C, E380Q) that are clinically relevant in ER+ breast cancer. Therefore, *ESR1*-specific false positives attributable to CHIP are biologically implausible. Nonetheless, when broad NGS panels are used and ambiguous low-VAF calls arise in non-*ESR1* genes, matched white blood cell (buffy coat) sequencing or germline filtering should be employed to exclude hematopoietic-origin variants.

Polyclonality is common and clinically significant. Multiple concurrent *ESR1* mutations are detected in 26–36% of *ESR1*-mutant cases, with 68.8% showing polyclonal patterns in serial monitoring studies [33, 37, 53]. Different *ESR1* variants may respond differently to specific SERDs, and shifting clonal dominance over time complicates single-agent strategies [5, 37].

ctDNA-tissue discordance has predictable patterns. ctDNA can be falsely negative when tumor shedding is low, particularly in limited-volume disease or non-visceral metastatic patterns [48, 49]. Conversely, ctDNA may detect *ESR1* mutations absent in a single tissue biopsy due to spatial heterogeneity or sampling of different metastatic clones [7, 47]. In patients with high clinical suspicion for endocrine resistance but negative ctDNA, tissue testing from an accessible progressing site should be considered [6, 45].

## Therapeutic targeting of *ESR1* mutations: from detection to clinical decision-making

The emergence of oral SERDs has transformed *ESR1*-mutant breast cancer from a biomarker-defined subgroup into a therapeutically actionable population. Meta-analyses demonstrate that oral SERDs improve PFS specifically in *ESR1*-mutant disease (HR 0.56, 95% CI 0.44–0.71), with no significant benefit in *ESR1* wild-type tumors (HR 0.94, 95% CI 0.78–1.14), establishing mutation status as a predictive rather than merely prognostic marker [65, 66] (Table 2).

**Table 2:** **Clinical trials of oral SERDs and ctDNA-guided switching strategies in *ESR1*-mutant ER+/HER2**– **advanced breast cancer.**

**Clinical trial**	**Agent**	**Population**	**Intervention arm**	**Comparator arm**	**Median PFS (*ESR1*-mut)**	**HR (95% CI)**	**Key findings**	**References**
**Classical trials: testing at progression**								
EMERALD	Elacestrant	Post-CDK4/6i progression; ER+/HER2– MBC	Elacestrant	SOC ET (fulvestrant or AI)	3.6 vs. 1.9 months	0.41 (0.26–0.63)	First FDA-approved oral SERD for *ESR1*-mut; benefit enriched with ≥ 12 months prior CDK4/6i (8.6 vs. 1.9 months)	[18]
EMBER-3	Imlunestrant	Post-AI ± CDK4/6i	Imlunestrant monotherapy or imlunestrant + abemaciclib	SOC ET (fulvestrant or exemestane)	10.9 vs. 5.5 months (imlunestrant–abemaciclib vs. imlunestran)	0.59 (0.47–0.74)	mOS 34.5 vs. 23.1 months (HR 0.60) in *ESR1*-mut; combination with abemaciclib improved PFS regardless of *ESR1* status	[67]
SERENA-2 (phase 2, non-registrational)	Camizestrant	Post-ET progression	Camizestrant (75 mg or 150 mg)	Fulvestrant	6.3 vs. 2.2 months	0.55 (0.33–0.91)	Phase 2: both 75 mg and 150 mg doses superior to fulvestrant; supported dose selection for SERENA-6	[16, 72]
VERITAC-2	Vepdegestrant	Post-CDK4/6i progression	Vepdegestrant 200 mg daily (oral)	Fulvestrant 500 mg IM	5.0 vs. 2.1 months	0.58 (0.43–0.78)	First phase 3 PROTAC ER degrader trial; primary endpoint met in *ESR1*-mut population only; ORR 18.6% vs. 4.0%; NDA submitted to FDA	[68]
persevERA	Giredestrant + palbociclib	Treatment-naïve, first-line; ER+/HER2–– LA/MBC	Giredestrant + palbociclib	Letrozole + palbociclib	Not reported	Not reported	Negative trial; numerical but not statistically significant PFS improvement in ITT population; *ESR1*-mut subgroup data and full results pending presentation	NCT04546009
**Interception trials: ctDNA-guided switching ahead of progression**								
SERENA-6	Camizestrant switch (double-blind, placebo-controlled)	*ESR1* emergence during first-line therapy AI + CDK4/6i; no clinical/radiologic progression	Camizestrant 75 mg + CDK4/6i + placebo for AI	Continue AI + CDK4/6i + placebo for camizestrant	16.0 vs. 9.2 months	0.44 (0.31–0.60)	First global registrational ctDNA-guided switching trial; only double-blind, placebo-controlled interception trial; 56% relative reduction in progression/death risk	[24]
PADA-1	Fulvestrant switch	*ESR1* emergence during first-line therapy AI + palbociclib; no clinical/radiologic progression	Switch to fulvestrant + palbociclib	Continue AI + palbociclib	11.9 vs. 5.7 months (fulvestrant and palbociclib group vs. AI and palbociclib group)	0.61 (0.43–0.86)	Proof-of-concept for ctDNA-guided switching; first trial to demonstrate PFS benefit from early therapy switch guided by liquid biopsy	[13]

AI: aromatase inhibitor; CDK4/6i: cyclin-dependent kinase 4 and 6 inhibitors; ctDNA: circulating tumor DNA; ER: estrogen receptor; ET: endocrine therapy; FDA: Food and Drug Administration; HER2: human epidermal growth factor receptor 2; HR: hazard ratio; IM: intramuscular; ITT: intent-to-treat; LA/MBC: locally advanced/metastatic breast cancer; mOS: median overall survival; NDA: new drug application; ORR: objective response rate; PFS: progression-free survival; PROTAC: proteolysis-targeting chimera; SERD: selective estrogen receptor degrader; SOC: standard of care.

Elacestrant became the first U.S. Food and Drug Administration (FDA)-approved oral SERD specifically indicated for *ESR1*-mutant ER+/HER2– advanced breast cancer following the EMERALD trial, which demonstrated median PFS of 3.8 vs. 1.9 months compared with standard endocrine therapy (HR 0.55, 95% CI 0.39–0.77; *P* = 0.0005) [19, 22]. Notably, benefit was most pronounced in patients with ≥ 12 months prior CDK4/6i exposure, where median PFS reached 8.6 vs. 1.9 months (HR 0.41) [18]. Real-world data confirm these findings, with a median time-to-next-treatment of 7.9 months across 306 patients [66]. However, the OS endpoint was not met in EMERALD; the FDA approval summary reported an OS HR of 0.90 (95% CI 0.63–1.30) in the *ESR1*-mutant subgroup, with no statistically significant OS benefit [22].

- Inluriyo (imlunestrant) received FDA approval in September 2025 based on EMBER-3, demonstrating a median PFS of 5.5 vs. 3.8 months in *ESR1*-mutant patients (*P* < 0.001) [25, 67]. Updated analyses show median overall survival (mOS) of 34.5 vs. 23.1 months (HR 0.60, 95% CI 0.43–0.86), though the prespecified significance boundary was not crossed [67]. The combination of imlunestrant with abemaciclib significantly improved PFS regardless of *ESR1* status (median 10.9 vs. 5.5 months; HR 0.59) [25, 67]. It should be noted that the FDA approval of Inluriyo (imlunestrant) was for monotherapy in *ESR1*-mutant disease; the combination of imlunestrant with abemaciclib did not receive FDA approval.
- Camizestrant demonstrated superiority over fulvestrant in SERENA-2 (median PFS 7.2–7.7 vs. 3.7 months), with particular benefit in *ESR1*-mutant tumors (6.3 vs. 2.2 months) [16]. The SERENA-6 trial then established the molecular interception paradigm: patients switched to camizestrant upon ctDNA-detected *ESR1* emergence (before radiographic progression) achieved median PFS of 16.0 vs. 9.2 months (HR 0.44, 95% CI 0.31–0.60; *P* < 0.0001) [24]. This represents the first global phase 3 trial demonstrating the clinical utility of ctDNA-guided therapy switching ahead of disease progression.
- Vepdegestrant, a PROTAC-based ER degrader, showed median PFS of 5.0 vs. 2.1 months in *ESR1*-mutant patients (HR 0.58; *P* < 0.001), with objective response rates of 18.6% vs. 4.0% [68]. The VERITAC-2 trial results showed that in the overall (unselected) population, the PFS difference did not reach statistical significance (HR 0.83, 95% CI 0.69–1.01; *P* = 0.07), confirming the predictive value of *ESR1* mutation status. Vepdegestrant was generally well tolerated, with treatment-related adverse event discontinuation rates of 2.9% vs. 0.7% for fulvestrant [68].

Co-occurring alterations influence treatment sequencing. Approximately 10–15% of patients harbor concurrent *ESR1* and *PIK3CA* mutations [69]. For patients with dual *ESR1* and *PI3K*-pathway alterations, real-world data suggest elacestrant monotherapy achieves a median time-to-next-treatment of 5.2–6.3 months, comparable to outcomes in phase 3 studies [66, 69]. The National Comprehensive Cancer Network (NCCN) guidelines recommend capivasertib plus fulvestrant for patients with *PIK3CA*, *AKT1*, or *PTEN* alterations after CDK4/6i progression, while elacestrant or imlunestrant are options for *ESR1*-mutant disease [62]. The optimal sequencing of these targeted approaches remains an active area of investigation [70, 71]. Mechanistically, *ESR1*-mutant tumors may upregulate PI3K/AKT signaling as a compensatory survival pathway, creating a molecular rationale for combination targeting. However, the choice between a SERD-based approach (for *ESR1*-mutant disease) and a PI3K/AKT pathway inhibitor-based approach (e.g., capivasertib + fulvestrant for PIK3CA/AKT1/PTEN-altered tumors) in the second-line setting after CDK4/6i progression is currently mutually exclusive in clinical practice [72]. Given that endocrine therapy-based third lines remain rare, patients are unlikely to receive both agents sequentially, making the initial therapeutic decision after CDK4/6i progression a critical and potentially irreversible choice. Prospective head-to-head data comparing SERD-first vs. PI3K inhibitor-first sequencing in dual-mutant disease are lacking and represent a key unmet research need.

## Conclusions

*ESR1* mutations represent a paradigm-defining example of how molecular oncology can transform clinical practice—from mechanistic discovery through biomarker-guided intervention to regulatory approval of mutation-specific therapeutics. These LBD alterations, rare in treatment-naïve tumors but emerging in 20–40% of patients following AI exposure, drive constitutive ER activation that renders continued estrogen deprivation ineffective while preserving sensitivity to receptor degradation strategies. The convergence of ctDNA technology with serial monitoring has reframed endocrine resistance as a dynamic, measurable evolutionary process rather than a binary clinical event, enabling detection of *ESR1*-mutant clones months before radiographic progression. Randomized evidence from PADA-1 and SERENA-6 demonstrates that therapeutic intervention triggered by molecular emergence (rather than delayed until clinical progression) significantly improves PFS, establishing ctDNA-guided switching as a clinically validated strategy. The regulatory approval of elacestrant specifically for *ESR1*-mutant disease, followed by imlunestrant, marks a transition from empirical endocrine sequencing to genotype-directed therapy selection. As oral SERDs, PROTAC-based degraders, and combination strategies continue to mature, the remaining challenges are operational rather than conceptual: standardizing thresholds for “rising” mutations, integrating polyclonality into treatment algorithms, and optimizing sequencing with PI3K/AKT pathway inhibitors in patients harboring concurrent alterations. The *ESR1* story thus provides a template for precision oncology in solid tumors, demonstrating that when mechanism, measurement, and therapeutic targeting align, resistance can be intercepted rather than merely observed. However, several important limitations should be acknowledged. OS data from the interception trials (PADA-1, SERENA-6) remain immature, and the EMERALD trial did not demonstrate a statistically significant OS benefit for elacestrant. The cost and infrastructure requirements of serial ctDNA monitoring present substantial barriers to global implementation, particularly in resource-limited settings. Furthermore, the mutually exclusive nature of current second-line therapeutic options (SERD vs. PI3K pathway inhibitor) in patients with dual alterations highlights a critical gap in evidence-based sequencing strategies. These operational and clinical challenges must be addressed to fully realize the promise of ESR1-guided precision oncology.

## Abbreviations

AI: aromatase inhibitor
ASCO: American Society of Clinical Oncology
CDK4/6: cyclin-dependent kinase 4 and 6
CDK4/6i: cyclin-dependent kinase 4 and 6 inhibitors
cfDNA: cell-free DNA
CHIP: clonal hematopoiesis of indeterminate potential
CI: confidence interval
ctDNA: circulating tumor DNA
ddPCR: droplet digital PCR
ER+: estrogen receptor-positive
FDA: Food and Drug Administration
HER2: human epidermal growth factor receptor 2
HR: hazard ratio
LBD: ligand-binding domain
MAF: mutant allele frequency
mOS: median overall survival
NGS: next-generation sequencing
PFS: progression-free survival
PI3K/AKT: phosphatidylinositol 3-kinase/protein kinase B
PROTAC: proteolysis-targeting chimera
SERDs: selective estrogen receptor degraders
VAF: variant allele frequency

## Declarations

### Copyright

© The Author(s) 2026.
